# Long-term efficacy and advantages of minimally invasive hepatectomy for hepatolithiasis: A protocol for systematic review and meta-analysis

**DOI:** 10.1097/MD.0000000000023230

**Published:** 2020-12-11

**Authors:** Jianyang Liu, Jinchai Xu, Dengpan Luo, Yujun Zhao, Hongbo Shen, Jianzhong Rao

**Affiliations:** a Quzhou People's Hospital; b Quzhou Hospital of Traditional Chinese Medicine, Quzhou; c Jiangshan People's Hospital, Jiangshan, Zhejiang Province, China.

**Keywords:** hepatolithiasis, meta-analysis, minimally invasive hepatectomy, protocol, systematic review

## Abstract

**Background:**

Hepatolithiasis commonly occurs in the bile duct proximal to the confluence of the right and left hepatic ducts, regardless of the coexistence of gallstones in gallbladder or the common bile duct. Clinical research proves that minimally invasive surgery is effective in the treatment of hepatolithiasis. Although previous meta-analysis also shows that it could reduce intraoperative bleeding and blood transfusion, and shorten hospital stay time, there are few meta-analyses on its long-term efficacy. We conducted the meta-analysis and systematic review to systematically evaluate the long-term efficacy and advantages of minimally invasive hepatectomy in the treatment of hepatolithiasis.

**Methods:**

Articles of randomized controlled trials will be searched in the PubMed, Medline, Embase, Cochrane Library, China National Knowledge Infrastructure, Chongqing VIP Chinese Science and Technology Periodical Database, Chinese Biological and Medical database, and Wanfang database until September, 2020. Literature extraction and risk of bias assessment will be completed by 2 reviewers independently. Statistical analysis will be conducted in RevMan 5.3.

**Results:**

This study will summarize the present evidence by exploring the long-term efficacy and advantages of minimally invasive hepatectomy in the treatment of hepatolithiasis

**Conclusions:**

The findings of the study will help to determine potential long-term efficacy and advantages of minimally invasive hepatectomy in the treatment of hepatolithiasis.

**Ethics and dissemination:**

The private information from individuals will not be published. This systematic review also will not involve endangering participant rights. Ethical approval is not required. The results may be published in a peer-reviewed journal or disseminated in relevant conferences.

**OSF Registration number:**

DOI 10.17605/OSF.IO/H6WRV.

## 1 Introduction

Hepatolithiasis commonly occurs in the bile duct proximal to the confluence of the right and left hepatic ducts, regardless of the coexistence of gallstones in gallbladder or the common bile duct.^[1–3]^ The incidence rate is 3.1% to 21.2%, mostly in Asia-Pacific region, such as China, Japan, and Korea.^[4]^ The obstruction caused by stone could lead to bile duct inflammation, stenosis, and liver fibrosis, and it may even further cause liver atrophy or malignant transformation to lead to serious complications of biliary tract and the whole system, which is the common cause of death in nonmalignant diseases of biliary tract.^[5]^

At present, the principal treatment for hepatolithiasis is hepatectomy,^[3]^ which could not only resect the lesion, but also could treat and prevent the complications of hepatolithiasis in the middle and late stages. Conventionally, open hepatectomy is frequently applied in the treatment of hepatolithiasis. Though it is a mature surgery, the prognosis is not obvious. After open surgery, the wound would be prone to get infected, leading to a variety of complications. Also, the repeated operations because of the recurrence could lead to reinfection, cholangitis, and other complications.^[6]^

With the development of minimally invasive surgery in recent years, laparoscopic and robot-assisted hepatectomy in the treatment of hepatolithiasis have gradually shown more advantages,^[2,7–9]^ such as less surgical trauma, less blood loss, faster postoperative recovery, minimal invasion.^[10]^ Clinical research proves that minimally invasive surgery is effective in the treatment of hepatolithiasis, and the operation time is shorter, with lower incidence of postoperative complications. Although previous meta-analysis also shows that minimally invasive hepatectomy could reduce intraoperative bleeding and blood transfusion, and shorten hospital stay time,^[11,12]^ there are few meta-analyses on its long-term efficacy. Therefore, to systematically evaluate the long-term efficacy and advantages of minimally invasive hepatectomy in the treatment of hepatolithiasis, we conducted the meta-analysis and systematic review.

## 2 Methods

### 2.1 Study registration

This protocol of systematic review and meta-analysis has been drafted under the guidance of the preferred reporting items for systematic reviews and meta-analyses protocols.^[13]^ Moreover, it has been registered on open science framework (OSF) on October 11, 2020. (Registration number: DOI 10.17605/OSF.IO/H6WRV)

### 2.2 Ethics

Ethical approval is not required for no patient enrolled and personal information collected, and the data are all derived from published studies.

### 2.3 Inclusion criteria for study selection

#### 2.3.1 Type of studies

Randomized controlled trials (RCTs) of minimally invasive hepatectomy in the treatment of hepatolithiasis will be included, and only literatures in Chinese and English are included.

#### 2.3.2 Type of participants

All the included participants conform to the diagnosis of hepatolithiasis, regardless of nationality, race, age, gender, and source.

#### 2.3.3 Type of interventions

The study focuses on RCTs of minimally invasive hepatectomy versus open hepatectomy in the treatment of hepatolithiasis, and the type of invasive hepatectomy will not be limited.

#### 2.3.4 Type of outcome measures

Perioperative outcomes include operative time, estimated blood loss, intraoperative transfusion, postoperative length of hospital stay, and postoperative complications.

Long-term outcomes are stone clearance and recurrence, cholangitis recurrence, the need for reoperation, morbidity, and mortality.

### 2.4 Exclusion criteria

1. Studies with unsatisfactory outcome indicators, or studies that did not completely describe the outcomes of interest;
2. Duplicated published literatures;
3. Extracting the related data from the published results is impossible, and unable to obtain the literatures after contacting the author;
4. Literatures with errors in random methods.

### 2.5 Search strategy

Articles of RCTs will be searched in the PubMed, Medline, Embase, Cochrane Library, China National Knowledge Infrastructure, Chongqing VIP Chinese Science and Technology Periodical Database, Chinese Biological and Medical database, and Wanfang database until Sep, 2020. The search terms are the following words in various combinations: “hepatolithiasis,” “intrahepatic stones,” “laparoscopy,” “minimally invasive surgery,” “liver resection,” and “hepatectomy.” The search strategy of PubMed is listed in Table 1.

**Table 1:** Search strategy in PubMed database.

Number	Search terms
#1	Hepatolithiasis [Title/Abstract]
#2	Intrahepatic bile duct stone [Title/Abstract]
#3	Hepatolith [Title/Abstract]
#4	Calculus of intrahepatic duct [Title/Abstract]
#5	#1 OR #2 OR #3 OR #4
#6	Minimally Invasive Surgical Procedures [MeSH Terms]
#7	Surgical Procedures, Minimal [Title/Abstract]
#8	Minimal Surgical Procedure [Title/Abstract]
#9	Laparoscope [Title/Abstract]
#10	Laparoscopic [Title/Abstract]
#11	da Vinci robot [Title/Abstract]
#12	#6 OR #7 OR #8 Or #9 OR #10 OR #11
#13	Hepatectomy [Title/Abstract]
#14	Hepatectomies [Title/Abstract]
#15	Liver resection [Title/Abstract]
#16	#13 OR #14 OR #15
#17	#5 AND #12 AND #16

### 2.6 Data extraction

**Figure 1:**
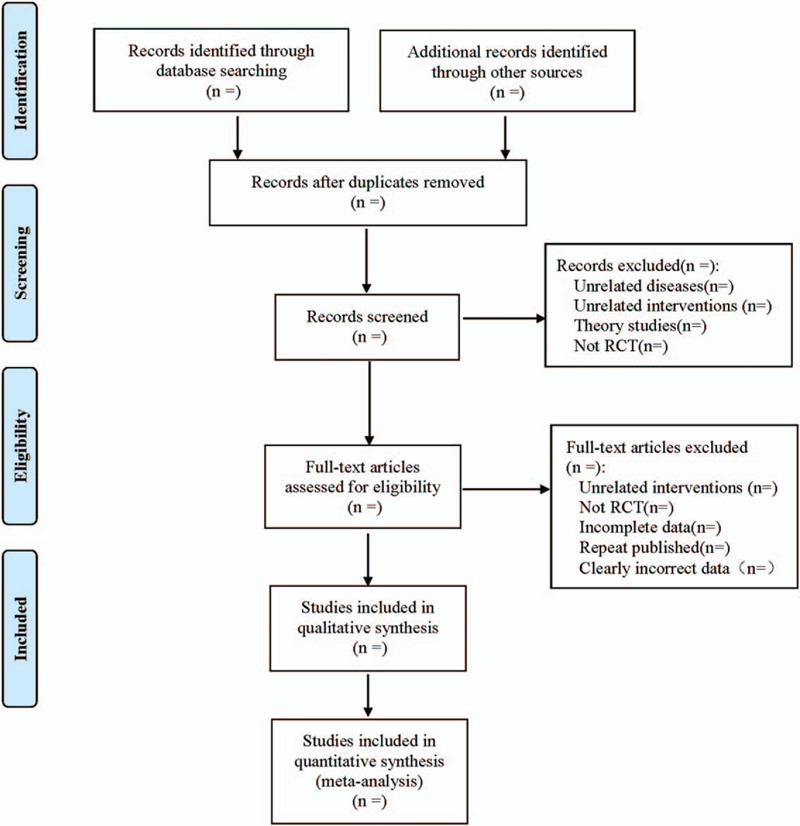
Flow diagram.

Literature management will be performed by Endnote X7, and the literature screening process is shown in Figure 1. Literature extraction will be completed by 2 reviewers in Excel 2019 with an extraction table, including study identification (title, authors, journal, publication year, and country), demographics, randomization, concealment, interventions, outcomes, adverse events. Discrepancies will be resolved by the senior author.

### 2.7 Risk of bias assessment

The risk of bias in RCTs will be independently evaluated by 2 reviewers in accordance with the Cochrane Handbook of Systematic Reviewers, including random sequence generation, allocation concealment, blinding of participants and outcome assessment, incomplete outcome data, selective reporting, and other bias. The qualities of studies will be evaluated using the Newcastle-Ottawa Quality Assessment Scale. In case of disagreement, a third senior will be consulted.

### 2.8 Statistical analysis

#### 2.8.1 Data synthesis

The statistical analysis will be performed by RevMan 5.3 (Cochrane Collaboration, Oxford, United Kingdom). The relative risk with the 95% confidence interval (CI) will be applied for dichotomous variables; weighted mean difference with the 95% CI is selected with different tools or units of measurement for continuous variables. Heterogeneity test was evaluated with Q test and quantified with the I^2^ statistic. It will be considered as statistical heterogeneity, and the random-effect model will be used for analysis if there is no obvious clinical or methodological heterogeneity.

#### 2.8.2 Dealing with missing data

Contact the corresponding author to get the missing data when data is missing or incomplete in a study. If unable to get in touch, the study will be abandoned.

#### 2.8.3 Subgroup analysis

Subgroup analysis is conducted according to type of operation, such as left lateral sectionectomy, left hemihepatectomy, and right hepatectomy.

#### 2.8.4 Sensitivity analysis

A one-by-one elimination method will be adopted for sensitivity analysis to test the stability of meta-analysis results of indicators.

#### 2.8.5 Reporting bias

Funnel plot will be used to qualitatively detect publication bias if the included study is more than 10 for the major outcome indicators. Potential publication bias will be quantitatively assessed by Egger and Begg test.

#### 2.8.6 Evidence quality evaluation

The quality of evidence will be assessed by the Grading of Recommendations Assessment, Development, and Evaluation with bias risk, consistency, directness, precision, and publication bias, rating as high, moderate, low, and very low.

## 3 Discussion

Hepatolithiasis is mainly caused by bacterial infection, parasites, bile stasis, anatomical variation, dietary structure, and genetics, with the pathological changes of hepatobiliary cholestasis, acute and chronic inflammation, inflammatory stenosis, and proximal dilation.^[14,15]^ The accumulating stones in dilated bile ducts could further aggravate the obstruction, leading to repeated inflammation, abscesses, systemic sepsis, and even biliary ulceration. Hepatectomy is the main method to remove stones, correct bile duct stenosis, smooth bile duct drainage, and prevent disease recurrence. The conventional open hepatectomy and the emerging minimally invasive surgery are believed to be different in intraoperative blood loss, intestinal function recovery time, hospital stay, and postoperative complications,^[5,16,17]^ which will also be evaluated by different meta-analysis.^[12,14,18,19]^ However, in addition to surgical risks and short-term postoperative complications, long-term postoperative complications, stone clearance and recurrence, cholangitis recurrence, the need for reoperation, morbidity, and mortality are also important indicators for evaluating the efficacy.

Therefore, in this study, we try to conduct this meta-analysis and systematic review to evaluate the long-term efficacy and advantages of minimally invasive hepatectomy in the treatment of hepatolithiasis. Yet, the study has following limitations: the included studies are written in Chinese or English, which may result in certain selective bias, and there might be some heterogeneity due to different minimally invasive hepatectomy techniques.

## Author contributions

**Conceptualization:** Dengpan Luo, Yujun Zhao.

**Data curation:** Jianyang Liu, Jinchai Xu.

**Investigation:** Dengpan Luo, Hongbo Shen.

**Resources:** Yujun Zhao.

**Software:** Hongbo Shen.

**Supervision:** Jianzhong Rao.

**Writing – original draft:** Jianyang Liu, Jinchai Xu.

**Writing – review & editing:** Jianzhong Rao.
